# HIF2alpha-Associated Pseudohypoxia Promotes Radioresistance in Pheochromocytoma: Insights from 3D Models

**DOI:** 10.3390/cancers13030385

**Published:** 2021-01-21

**Authors:** Verena Seifert, Susan Richter, Nicole Bechmann, Michael Bachmann, Christian G. Ziegler, Jens Pietzsch, Martin Ullrich

**Affiliations:** 1 Department of Radiopharmaceutical and Chemical Biology, Helmholtz-Zentrum Dresden-Rossendorf, Institute of Radiopharmaceutical Cancer Research, 01328 Dresden, Germany; v.seifert@hzdr.de; 2 Faculty of Chemistry and Food Chemistry, School of Science, Technische Universität Dresden, 01069 Dresden, Germany; 3 Institute of Clinical Chemistry and Laboratory Medicine, University Hospital Carl Gustav Carus at Technische Universität Dresden, 01307 Dresden, Germany; susan.richter@uniklinikum-dresden.de (S.R.); nicole.bechmann@uniklinikum-dresden.de (N.B.); 4 Department of Internal Medicine III, University Hospital Carl Gustav Carus at Technische Universität Dresden, 01307 Dresden, Germany; christian.ziegler@uniklinikum-dresden.de; 5 Department of Experimental Diabetology, German Institute of Human Nutrition Potsdam-Rehbruecke, 14558 Nuthetal, Germany; 6 German Center for Diabetes Research (DZD), 85764 München-Neuherberg, Germany; 7 Department of Radioimmunology, Helmholtz-Zentrum Dresden-Rossendorf, Institute of Radiopharmaceutical Cancer Research, 01328 Dresden, Germany; m.bachmann@hzdr.de

**Keywords:** paraganglioma, radionuclide therapy, lutetium-177, spheroid control dose, SCD_50_, spheroid re-growth, irradiation, X-ray, radioresistance

## Abstract

**Simple Summary:**

Low oxygen levels (hypoxia) as well as genetic defects activating hypoxia signaling pathways (pseudohypoxia) are known to contribute to tumorigenesis and therapy resistance in various cancers. The genetic background of pheochromocytomas and paragangliomas is well characterized and indicates that pseudohypoxia plays a role in tumor formation and metastatic spread in a subgroup of these tumors. It is, however, unknown how pseudohypoxia affects susceptibility to radiation treatments, which is of particular interest, since targeted radionuclide therapy is one of the few options used against metastatic pheochromocytomas and paragangliomas. To date, no curative treatment is available for metastatic disease. Here, we report on the radioprotective effects of pseudohypoxia against both external irradiation and beta particle-emitting lutetium-177 in a pheochromocytoma tumor spheroid model expressing hypoxia-inducible factor 2 alpha. Our findings highlight hypoxia signaling pathways as potential targets for neo-adjuvant—in particular, radiosensitizing—therapies in pseudohypoxic pheochromocytomas and paragangliomas.

**Abstract:**

Pheochromocytomas and paragangliomas (PCCs/PGLs) are rare neuroendocrine tumors arising from chromaffin tissue located in the adrenal or ganglia of the sympathetic or parasympathetic nervous system. The treatment of non-resectable or metastatic PCCs/PGLs is still limited to palliative measures, including somatostatin type 2 receptor radionuclide therapy with [^177^Lu]Lu-DOTA-TATE as one of the most effective approaches to date. Nevertheless, the metabolic and molecular determinants of radiation response in PCCs/PGLs have not yet been characterized. This study investigates the effects of hypoxia-inducible factor 2 alpha (HIF2α) on the susceptibility of PCCs/PGLs to radiation treatments using spheroids grown from genetically engineered mouse pheochromocytoma (MPC) cells. The expression of *Hif2α* was associated with the significantly increased resistance of MPC spheroids to external X-ray irradiation and exposure to beta particle-emitting [^177^Lu]LuCl_3_ compared to *Hif2α*-deficient controls. Exposure to [^177^Lu]LuCl_3_ provided an increased long-term control of MPC spheroids compared to single-dose external X-ray irradiation. This study provides the first experimental evidence that HIF2α-associated pseudohypoxia contributes to a radioresistant phenotype of PCCs/PGLs. Furthermore, the external irradiation and [^177^Lu]LuCl_3_ exposure of MPC spheroids provide surrogate models for radiation treatments to further investigate the metabolic and molecular determinants of radiation responses in PCCs/PGLs and evaluate the effects of neo-adjuvant—in particular, radiosensitizing—treatments in combination with targeted radionuclide therapies.

## 1. Introduction

Pheochromocytomas and paragangliomas (PCCs/PGLs) are rare neuroendocrine tumors arising from the adrenal medulla or from extra-adrenal chromaffin tissue, respectively, with an incidence of between 2 and 8 cases per 1 million per year [1]. PCCs/PGLs are predominantly benign [2,3]; however, 10% are at risk of tumor relapse after surgical resection [4]. For non-resectable or metastatic PCCs/PGLs, all currently available treatment options are considered palliative [5,6].

The increased density of somatostatin type 2 receptors (SSTR2) in neuroendocrine tumors allows for peptide receptor radionuclide therapy. [^177^Lu]Lu-DOTA-TATE is one such therapy, and has shown encouraging treatment responses, including in PCCs/PGLs [7,8,9,10,11]. Radionuclide therapy is a fast-evolving treatment modality with a high potential for personalized cancer therapy. Compared to external beam radiotherapy, it is associated with a reduced risk of collateral tissue damage and less severe side effects [12,13]. Tumor relapse after initially good treatment response has been observed in patients with metastatic paraganglioma as well as in established murine tumor models of pheochromocytomas in vivo [14,15]. In order to improve therapy outcomes, a combination of targeted radionuclide therapy with neo-adjuvant or radiosensitizing therapies has been suggested [14,16,17,18]. Since a subgroup of PCCs/PGLs is characterized by elevated levels of hypoxia-inducible factors (HIFs), known to be associated with resistance to radiotherapy and chemotherapy in various cancers, hypoxia-related pathways may be considered as targets for neo-adjuvant therapies [19,20,21].

HIFs are key mediators of metabolic responses to hypoxia and are hence involved in regulating pathways relying on oxygen availability [22]. Under normoxic conditions, HIFα subunits are ubiquitin-tagged and degraded in proteasomes [22] after hydroxylation by prolyl hydroxylase domain proteins belonging to the Fe(II)- and 2-oxogluterate-dependent oxygenase superfamily [23]. Under hypoxic conditions, HIFα subunits remain stable and form active transcription factors by interacting with various cofactors such as HIFβ, c-Myc, or p53 [22]. In a subgroup of PCCs/PGLs, mutations of germline and somatic origin associated with the dysregulation of hypoxia signaling pathways have been described to result in the oxygen-independent stabilization of HIFα—a metabolic state that is referred to as pseudohypoxia [2,22]. Hypoxic and pseudohypoxic conditions are known to be associated with downstream transcriptional changes that eventually activate oncogenic pathways [21,24]. In PCCs/PGLs, the increased stabilization of the HIF2α isoform, encoded by the endothelial PAS domain-containing protein 1 (*EPAS*1) gene, has been reported to be associated with rapid tumor progression and worse prognosis [25,26], thus presenting itself as a promising target for future treatment approaches. Since the stabilization of HIF2α is often associated with treatment resistance in various cancers, we hypothesized that it may also contribute to the radiation resistance of PCCs/PGLs.

We recently reported on genetically engineered *Hif2α*-expressing mouse pheochromocytoma (MPC) cell spheroids as a three-dimensional in vitro model for investigations into the effects of dysregulated hypoxia signaling pathways in PCCs/PGLs [27,28]. To address the above hypothesis, the objectives of this study were (1) to establish robust methodologies for precisely characterizing the morphologic responses of MPC spheroids to radiation treatments, comparing single-dose X-ray irradiation with temporary exposure to [^177^Lu]LuCl_3_, and (2) to apply these methodologies with the purpose of determining the effects of *Hif2α* expression on the radiation response of MPC spheroids.

## 2. Results

### 2.1. Response of MPC^wt^ Spheroids to External X-ray Irradiation

**Figure 1:**
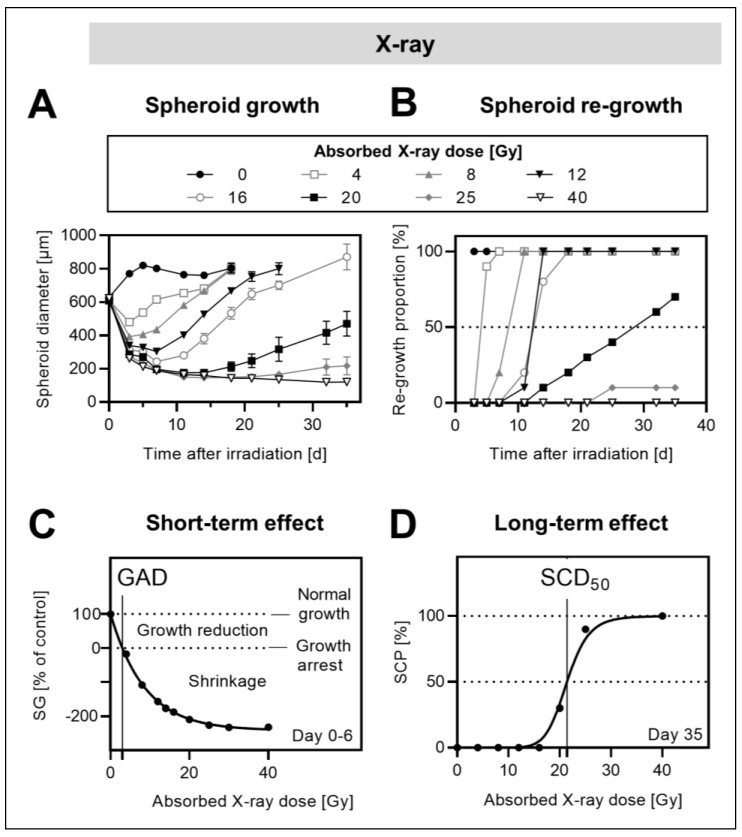
Response of MPC^wt^ spheroids to single-dose external X-ray irradiation; diameter at treatment start: 616 ± 2 µm (MPC^wt^); (**A**) changes in spheroid diameters in response to increasing irradiation doses; (**B**) spheroid re-growth proportions in treatment groups exposed to increasing irradiation doses; (**C**) short-term effects of irradiation at increasing doses presented as changes in relative spheroid growth (%SG, 0–6 days after irradiation), categorized into different growth scenarios and visualized as growth arrest dose (GAD); (**D**) long-term effects of irradiation at increasing doses presented as spheroid control probabilities (%SCP, 35 days after irradiation) and visualized as half-maximal spheroid control dose (SCD_50_).

The external irradiation (X-ray) of MPC^wt^ spheroids was investigated as a basic model for the radiation treatment of PCCs/PGLs. Single-dose exposure between 4 and 40 Gy (6–9 days after cultivation start) induced shrinkage, followed by either re-growth or disintegration, whereas non-treated spheroids grew continuously up to diameters of 873 ± 14 µm within two weeks after the cultivation start (Figure 1A). The shrinkage of treated spheroids occurred within the first 3 days after irradiation with negative growth rates between −43 ± 2 µm/d (4 Gy) and −118 ± 1 µm/d (40 Gy). The growth rates showed a negative linear relationship with the irradiation dose (Table 1).

The re-growth of treated spheroids during a 35-day follow-up period showed three different dose–effect scenarios (Figure 1B): (1) the re-growth of all spheroids with a dose-dependent delay (4–16 Gy), (2) the partial re-growth of spheroids with increasing fractions of disintegrated and therefore controlled spheroids in relation to dose (20–25 Gy), and (3) the sustained control of all spheroids per group (40 Gy).

In all further investigations, the effects of radiation treatments of spheroids were analyzed referring to the above-described dose–effect categories.

The short-term effects of irradiation on MPC^wt^ spheroids were analyzed by taking the 6-day relative spheroid growth (%SG, compared to controls) and reporting reduced growth at doses below the calculated growth arrest dose (GAD) of 3 ± 0.1 Gy and shrinkage at doses higher than the GAD (Figure 1C).

The long-term effects of irradiation on MPC^wt^ spheroids were analyzed in terms of the 35-day spheroid control probability (%SCP). For MPC^wt^ spheroids, this ranged from 30% to 100% for doses between 20 and 40 Gy (Figure 1D), with a resulting half-maximal spheroid control dose of 21 ± 0.3 Gy.

### 2.2. Effect of Hif2α Expression on Response of MPC Spheroids to External X-ray Irradiation

**Figure 2:**
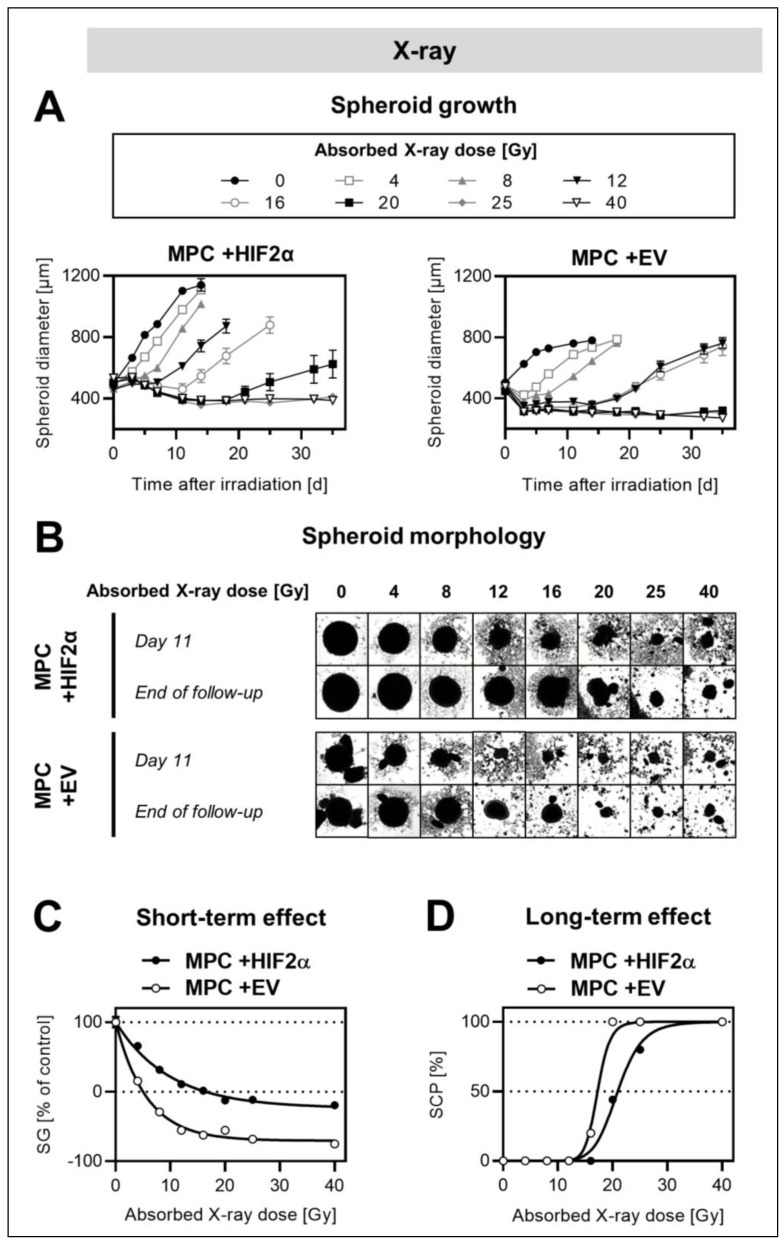
Effect of *Hif2α* expression on the response of MPC spheroids to single-dose external X-ray irradiation; diameters at treatment start: 499 ± 3 (MPC +HIF2α), 479 ± 3 (MPC + EV); (**A**) changes in spheroid diameters in response to increasing single-doses of irradiation; (**B**) arrays of spheroid micrographs recorded at day 11 compared to the final day of follow-up (each image shows a section of 1.57 × 1.57 mm^2^); (**C**) short-term effects of irradiation at increasing doses presented as relative spheroid growth (%SG, 0–6 days after irradiation); (**D**) long-term effects of irradiation at increasing doses presented as spheroid control probabilities (%SCP, 35 days after irradiation).

Genetically modified MPC +HIF2α spheroids showed increases in growth rates, maximum diameters, and resistance to external irradiation compared to MPC +EV spheroids, resembling a *Hif2α*-deficient “empty vector” control (Figure 2A). The expression of *Hif2α* impacted both the short-term and long-term effects of external irradiation.

Analyses of the short-term effects revealed that MPC +HIF2α spheroids initially maintained growth and showed a dose-dependent delay in shrinkage at ≥12 Gy, whereas, within the same dose range, MPC +EV spheroids shrank immediately, with 3-day negative growth rates between −24 ± 2 µm/day (4 Gy) and −66 ± 4 µm/day (40 Gy). The growth rates showed a negative linear relationship with irradiation doses in both cell lines (Table 1).

A significantly (*p* < 0.001) higher irradiation dose was necessary to achieve the growth arrest of MPC +HIF2α (GAD = 16 ± 1 Gy) compared to MPC +EV spheroids (GAD = 5.0 ± 0.3 Gy) (Figure 2B). At the highest irradiation dose of 40 Gy, the reduction in the relative growth of MPC +HIF2α spheroids (%SG = −19 ± 3%) was significantly (*p* < 0.001) lower compared to MPC +EV spheroids (%SG = −75 ± 4%).

In line with these observations, the evaluation of the long-term effects showed that significantly (*p* < 0.001) higher irradiation doses were necessary to achieve the sustained control of MPC +HIF2α spheroids (SCD_50_ = 21 ± 0.3 Gy) compared to MPC +EV spheroids (SCD_50_ = 17 ± 0.3 Gy) (Figure 2C). Spheroids, in particular of the MPC +HIF2α cell line, re-grew with dose-dependently reduced spherical symmetry (Figure S1).

### 2.3. Impact of G418 and DMSO on the Experimental Outcome of Spheroid Irradiation Treatment

Additional investigations on the supplementation of nutritional medium with frequently used cell culture additives showed that G418 (250 µg/mL) and DMSO (0.5%) significantly (*p* < 0.001) altered the treatment outcome after the external irradiation of both MPC +HIF2α and MPC +EV spheroids (for details see Supplementary Data, Table S3 and Figure S2). In brief, the radiosensitizing properties of G418 decreased the doses required for the long-term control of spheroids, whereas the radioprotective properties of DMSO increased the doses required for achieving both short-term growth arrest and long-term spheroid control.

### 2.4. Effects of Hif2α Expression on the Response of MPC Spheroids to Incubation with [^177^Lu]LuCl_3_

**Figure 3:**
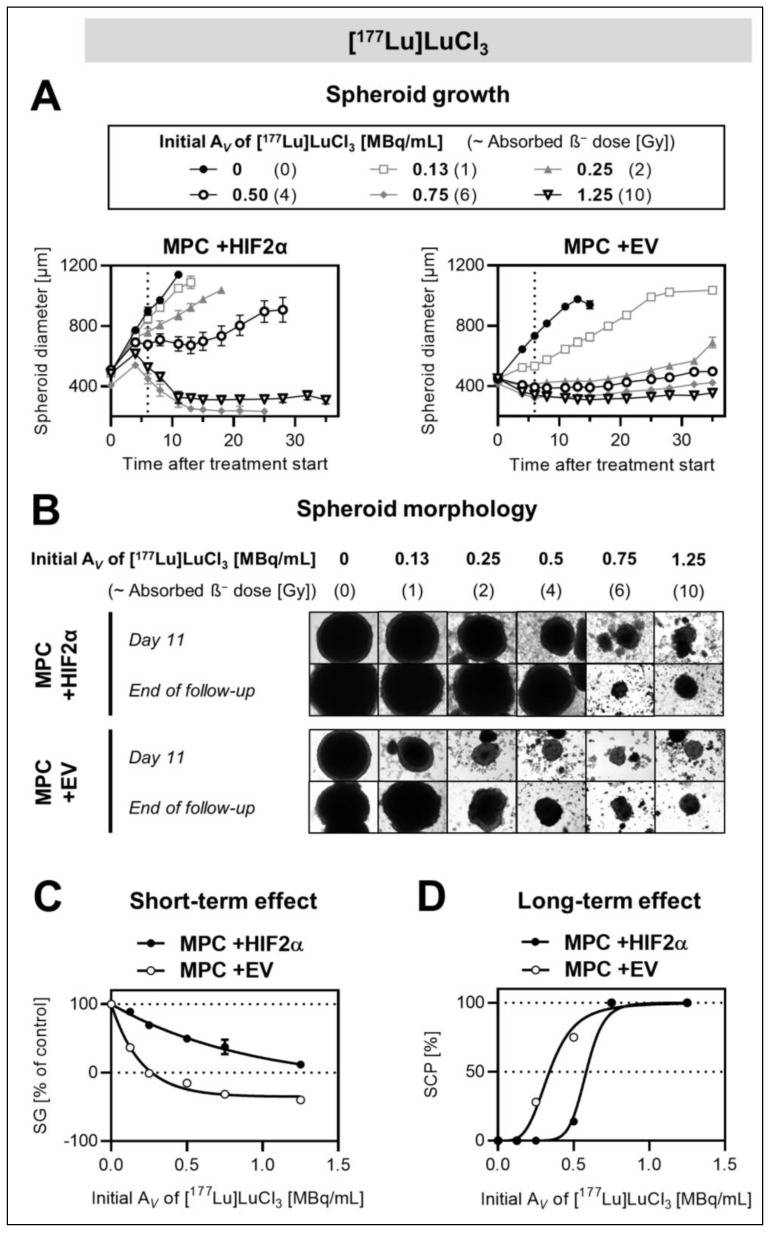
Effect of *Hif2α* expression on the response of MPC cell spheroids to [^177^Lu]LuCl_3_; diameters at treatment start: 492 ± 6 (MPC +HIF2α), 448 ± 3 (MPC + EV); (**A**) changes in spheroid diameters in response to increasing initial activity concentrations of [^177^Lu]LuCl_3_ (vertical dashed lines: end of treatment); (**B**) arrays of spheroid micrographs recorded at day 11 compared to the final day of follow-up (each image shows a section of 1.29 × 0.96 mm^2^); (**C**) short-term effects of [^177^Lu]LuCl_3_ with increasing initial activity concentrations presented as relative spheroid growth (%SG, 0–6 days after incubation start); (**D**) long-term effects of [^177^Lu]LuCl_3_ with increasing initial activity concentrations presented as spheroid control probabilities (%SCP, 35 days after treatment start).

Since single-dose external X-ray irradiation is not commonly applied as a treatment for inoperable PCCs/PGLs, a second radiation treatment approach based on a 6-day incubation with [^177^Lu]LuCl_3_, considered as a simplified model of radionuclide therapy, was investigated in our spheroid models. After exposure to initial activity, concentrations between 0 and 1.25 MBq/mL, which approximately correspond to absorbed doses between 0 and 10 Gy, both MPC +HIF2α and MPC +EV spheroids showed three response scenarios, similar to what has been observed after external irradiation (Figure 3A). MPC +HIF2α initially maintained growth; four days after treatment start, dose-dependent shrinkage with negative growth rates between −13 ± 13 µm/day (0.75 MBq/mL) and −40 ± 5 µm/day (1.25 MBq/mL) were observed. In comparison, the shrinkage of MPC +EV occurred immediately after treatment start at initial activity concentrations >0.25 MBq/mL (Table 1).

Short-term growth arrest was reached at significantly (*p* < 0.05) higher initial activity concentrations administered to MPC +HIF2α spheroids (GAD = 1.7 ± 0.7 MBq/mL, ≈14 ± 6 Gy) compared to MPC +EV (GAD = 0.3 ± 0.02 MBq/mL, ≈2.2 ± 0.2 Gy) (Figure 3B). At the highest initial activity concentration (1.25 MBq/mL, ≈10 Gy), the reduction in the relative growth of MPC +HIF2α spheroids (%SG = 12 ± 2%) was significantly (*p* < 0.001) less compared to that of MPC +EV spheroids (%SG = −40 ± 2%). The long-term control of MPC +HIF2α spheroids required higher initial activity concentrations (SCD_50_ = 0.6 ± 0.02 MBq/mL, ≈4.6 ± 0.2 Gy) compared to MPC +EV (SCD_50_ = 0.3 ± 0.02 MBq/mL, ≈2.7 ± 0.1 Gy), (Figure 3C).

In summary, [^177^Lu]LuCl_3_ exposure confirmed the results from the external irradiation experiments, in that *Hif2α* expression is associated with the increased radioresistance of MPC spheroids. Compared to external irradiation, however, exposure to [^177^Lu]LuCl_3_ required lower absorbed doses to achieve similar treatment effects (Table 2).

Additional experiments for he methodological validation of [^177^Lu]LuCl_3_ incubation as a model for radionuclide treatment showed that the position of spheroids across a 96-well microplate did not affect the experimental outcome (for details see Supplementary Figure S3). On the other hand, the spheroid size at treatment start (6–9 days after cultivation start) impacted the long-term response to [^177^Lu]LuCl_3_, as SCD_50_ increased with the initial spheroid diameter (Supplementary Figure S4, Table S5).

## 3. Discussion

In the present study, we successfully established robust methodologies for characterizing the growth responses of MPC spheroids after single-dose X-ray irradiation and after exposure to [^177^Lu]LuCl_3_. We successfully applied these methods to investigate the effects of *Hif2α* expression on the radiation response of MPC spheroids. Our investigations provide the first experimental evidence that the expression of *HIF2a* contributes to a radiation-resistant phenotype of PCCs/PGLs.

PCCs/PGLs of the pseudohypoxic cluster are characterized by higher *HIF2α* expression and HIF2α protein stabilization through multiple mechanisms, including mutations in *VHL*, *FH* or succinate dehydrogenase genes preventing HIF degradation or mutations sparing the protein from proteasomal degradation [25,29,30]. Since metastatic disease, for which one treatment choice is endoradiotherapy, more often occurs in pseudohypoxic PCCs/PGLs, our results are highly relevant for clinical decision-making with respect to underlying germline or somatic mutations [31].

Responses of tumor cells to ionizing radiation are frequently investigated in monolayer cell culture after treatment with single-dose external irradiation [32,33]. Treatment effects of external irradiation are then reported as clonogenic survival based on analyses of colony formation in cell cultures grown from single-cell suspensions [34,35]. The in vivo situation is, however, better reflected through the three-dimensional growth of tumor cells as spheroids [35,36,37]. The presence of stable hypoxic regions in spheroids makes it unnecessary to perform cultivation under extrinsic hypoxia that is often disturbed by temporary oxygen exposure during medium changes and microscopic documentation. Under normoxic conditions, radiation creates reactive oxygen specimens and oxygenated DNA radicals causing irreparable DNA strand breaks. Intrinsic hypoxia, as present in tumors and spheroids, prevents these formations resulting in inefficient DNA damage and radioresistance [20]. Genomic instability and inhibition of DNA repair pathways are herein mainly mediated by HIF1α [25]. The evaluation of radiation responses in spheroids requires alternative experimental readouts and analytical approaches as opposed to monolayer culture. In the present study, the evaluation of radiation treatment effects is based on the microscopic monitoring of spheroid diameters for up to 35 days after treatment start. The advantage of this data acquisition method is that both short-term and long-term treatment effects can be analyzed from one data set that has been recorded from the same spheroid sample.

Our approach to evaluating the short-term effects of radiation treatments builds on measuring dose-dependent changes in initial spheroid growth within six days after treatment start and on the calculation of the treatment dose required for effectively inducing growth arrest, referred to as the growth arrest dose (GAD). The exposure of spheroids to ionizing radiation at the GAD most likely induces clustered or sub-lethal DNA damage. Most of the viable cells within the spheroid undergo cell cycle arrest at the G2/M checkpoint in order to push resources towards DNA damage repair [38]. Shrinkage of spheroids exposed to ionizing radiation at doses higher than the GAD most likely occurred as a result of cluster-damaged cells undergoing necrosis or apoptosis. Therefore, initial changes in spheroid growth basically represent a change in size occurring due to radiation-induced cellular damage.

Our approach to evaluating the long-term effects of radiation treatments builds on determining the percentage of non-re-grown and therefore controlled spheroids after a follow-up of 35 days and on the calculation of the half-maximal spheroid control dose (SCD_50_). Sudden radiation-induced death of a considerable fraction of the cell population within a spheroid drastically improves the nutrient and oxygen supply of the surviving cells, thus reactivating previously quiescent cells [38]. Sub-lethally damaged cells that were able to finish DNA repair alongside non-damaged possibly re-activated cells proliferate and thereby repopulate the spheroid resulting in its re-growth. Therefore, the evaluation of spheroid control characterizes the surviving fraction of cells after radiation treatment, similar to colony formation assays.

As a first step towards establishing robust methodologies for investigations of the radiation responses of PCC/PGL spheroid models, the concept of simultaneously evaluating short-term and long-term effects was applied to MPC^wt^ spheroids treated with external X-ray irradiation at increasing single doses. Our investigations showed that the absorbed doses required for inducing short-term growth arrest were much lower compared to the doses required for achieving long-term spheroid control. These observations demonstrate that tumor cells are able to recover from much higher doses than short-term responses might suggest. Irradiation induces DNA damage with lower doses, causing mainly repairable single strand breaks, whereas higher doses also create double strand breaks, considered as largely irreparable [39]. Therefore, shrinkage followed by re-growth may result from the induction and repair of single-strand breaks, suggesting that spheroid control is a better indicator for treatment success compared to the growth arrest of spheroids.

Applying these methodologies to in vitro irradiation treatment approaches, we observed that *Hif2α*-expressing MPC cell spheroids were less sensitive to external X-ray irradiation than empty vector controls. The shrinkage of spheroids either occurred delayed by a few days or did not occur at all when treated with lower doses, whereas spheroids of control cells shrank immediately, similar to wild type. *Hif2α* expression enabled spheroids to re-grow after exposure to higher doses compared to control. As a result, *Hif2α*-expressing MPC spheroids were characterized by elevated doses necessary for short-term growth arrest and long-term control, suggesting that HIF2α contributes to a radioresistant phenotype of murine pheochromocytoma cells. These findings are in line with other reports showing that HIFs contribute to radioresistance in various cancers—e.g., by regulating the expression of tumor suppressor p53 [24,25]. In particular, HIF2α was found to be associated with radioresistance in renal cell carcinoma [24].

**Figure 4:**
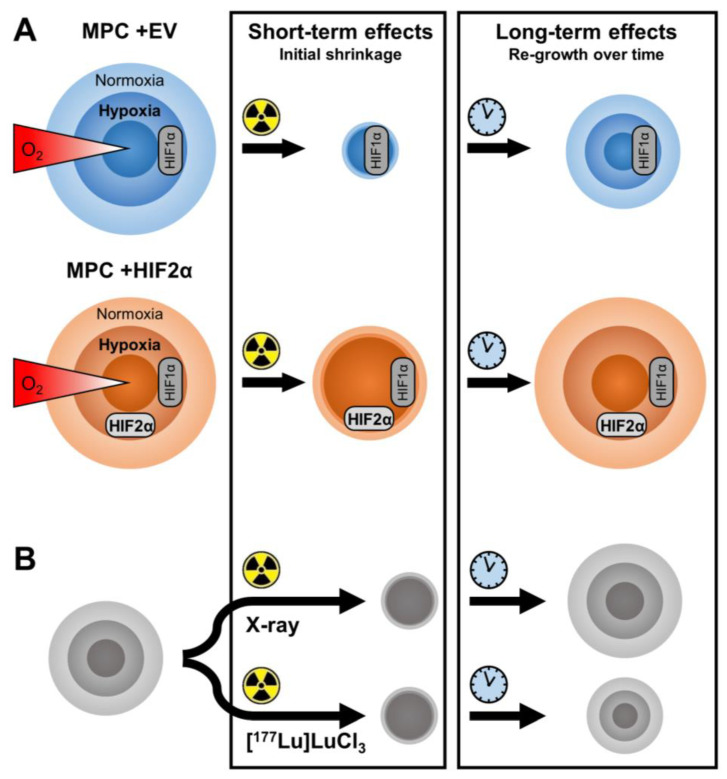
Response of MPC spheroids to radiation treatments. (**A**) Expression of *Hif2α* increases the radioresistance of MPC spheroids affecting both initial shrinkage and re-growth over time; (**B**) prolonged [^177^Lu]LuCl_3_ treatment more effectively delays the re-growth of MPC spheroids compared to single-dose external X-ray irradiation.

In agreement with previous reports, *Hif2α* expression in MPC spheroids increased the growth rates and maximum diameters [27]. These findings suggest an activation of growth-stimulating pathways through HIF2α in MPC cell spheroids, since growth saturation is a state characterized by equal rates of cell proliferation and death [37,38,40]. These results are in agreement with previous studies investigating HIF2α in the context of cell proliferation in tumors, including PCCs/PGLs [21,27,31,41,42,43]. In spheroids only, normoxic outer cell layers proliferate [38], suggesting that the effect in *Hif2α* expressing MPC spheroids is independent of oxygen levels and is thereby representing a pseudohypoxic state. The increased proliferative potential under normoxic conditions in these cell lines has been reported in previous studies [27,31,41], and might be explained by changes in the methylome and the ability of HIF2α to stabilize at near normoxic oxygen levels [42,44]. Therefore, in addition to the HIF1α-mediated intrinsic hypoxia prominent in both empty vector control and *Hif2α*-expressing MPC, HIF2α-mediated pseudohypoxia further enhances radioresistance in pheochromocytoma cells by protecting the more radiosensitive outer cell layers (Figure 4A).

*HIF2α* expression and protein stabilization is suspected to promote aggressive behavior and metastatic disease in PCCs/PGLs [31,43,45,46]. Since spheroid re-growth after irradiation is associated with increased growth rates compared to pre-treatment growth [38], the reduced spherical symmetry of re-grown spheroids of *Hif2α*-expressing MPC cells may reflect enhanced aggressive potential linked to HIF2α in recurring tumor cell clusters. The strongly reduced difference between external irradiation GAD and SCD_50_ in *Hif2α*-expressing MPC cell spheroids compared to controls may indicate the presence of HIF2α-mediated protective mechanisms against minor DNA and cell damage.

In the present study, G418 served as an antibiotic for the selection of genetically modified MPC cells in monolayer culture and was used only during routine passaging prior to spheroid formation. When G418 remained in the medium during treatment experiments instead of penicillin and streptomycin, MPC spheroids showed elevated radiosensitivity characterized by significantly decreased SCD_50_. For this reason, we recommend excluding G418 for irradiation studies of G418-selected cells. Since aminoglycosides are associated with nephrotoxicity, which is also the dose-limiting adverse effect of [^177^Lu]Lu-DOTA-TATE treatment, G418 cannot be considered as a potential radiosensitizer for clinical application [8,47,48].

Targeted radionuclide therapy is a radiotherapy approach considered to be superior to external irradiation, as it enables the selective targeting of tumor cells and is associated with reduced collateral tissue damage [13]. Lutetium-177 is currently the nuclide of choice for peptide receptor radionuclide therapy of neuroendocrine tumors—e.g., applied as [^177^Lu]Lu-DOTA-TATE [7,14]. In the present study, the treatment of MPC spheroids with [^177^Lu]LuCl_3_ as a simplified model of radionuclide therapy was more effective compared to single-dose external irradiation. This was particularly noticeable in the improvement of long-term effects with an SCD_50_ reduced by around 15 Gy (Figure 4B). The effect most likely results from prolonged radiation exposure during [^177^Lu]LuCl_3_ incubation, where spheroids re-oxygenate after initial radiation-induced tissue damage, leading to the enhanced radiosensitivity of previously radioresistant tumor cells [38]. The 6-day GAD resembling short-term effects of [^177^Lu]LuCl_3_ was only reduced by around 2.5 Gy compared to external irradiation, although the dose rate in radionuclide therapy is weaker compared to in external beam radiation therapy [32]. In our preclinical setup, both external irradiation and [^177^Lu]LuCl_3_ incubation detected the radioprotective effects of *Hif2α* expression in MPC spheroids and are therefore both suitable methodologies for characterizing changes in radiation response in vitro.

By using [^177^Lu]LuCl_3_ instead of, e.g., [^177^Lu]Lu-DOTA-TATE for the incubation of MPC spheroids, we excluded all aspects of SSTR2-specific targeting from interfering with the experimental outcome of radionuclide treatment. This was particularly important for our present investigation, since we previously reported the down-regulation of SSTR2 occurring in *Hif2α*-expressing MPC cells in vitro [27]. This observation is in line with *EPAS1*-mutated PCCs/PGLs presenting with reduced affinity to [^68^Ga]Ga-DOTA-TATE compared to other genetic variants [26,49].

The absorbed β^−^ dose in spheroids was estimated based on the initial activity concentration of [^177^Lu]LuCl_3_ irradiating the spheroid from the surrounding nutritional medium. This estimation excluded all aspects of activity distribution within the spheroid tissue. To our knowledge, there is no generally accepted calculation model available for estimating the absorbed β^−^ dose of spheroids irradiated by the surrounding medium in concave-bottom microtiter plates. As a percentage, the overall variation of our approximated doses compared to examples in the literature amounted to 7 ± 2% (Table S1), with higher variation for doses > 10 Gy (14 ± 4%, *n* = 4) but smaller variations for doses in our applied dose range of ≤10 Gy (4 ± 2%, *n* = 6) [32,33]. Our own calculations most closely resemble the sphere model predictions [33].

The different responses to [^177^Lu]LuCl_3_ incubation observed in spheroids of different initial size demonstrate that initial spheroid size strongly determines the treatment outcome in vitro. Increased radioresistance may partly be caused by more pronounced hypoxic regions and enhanced HIF1α-signaling. Similar effects are known from radiotherapy where tumor size at treatment start often determines treatment success and prognosis in patients [50,51]. Therefore, comparison of radiation effects between different treatment groups requires the precise matching of initial spheroid size.

One difficulty with evaluating the effects of radiation treatment on MPC spheroids was related to the correct measurement of the spheroid diameters. Irradiated spheroids accumulated debris in a cloud-like manner leading to contrast issues during the analysis of micrographs, thus making it sometimes difficult to differentiate whether a spheroid remained in growth arrest or had already disintegrated. Scans should therefore be revised manually, although, referring to the comparably long monitoring period and large amount of raw data, we highly recommend a fully or semi-automated graphical analysis for high throughput.

To further reduce inaccuracies in determining the spheroid control probabilities, re-growth was not only defined, as it usually is, by the threshold of spheroid size [52,53], but also by the slope of diameter–time plots. During growth arrest, changes in spheroid diameters showed a variation of up to 20 µm/day. Furthermore, disintegration sometimes appeared as an increase in diameter, however it showed a smaller growth rate compared to truly re-growing spheroids. We therefore used a diameter change of 20 µm/day as the slope threshold to define spheroid growth, even for small spheroids below a fixed diameter threshold.

Radiation treatment assays using MPC spheroids provide an experimental platform to screen for potential molecular determinants of radiation response as well as for radiation-induced targets to be addressed in neo-adjuvant therapies for PCCs/PGLs. The combination of radionuclide therapy with neo-adjuvant treatments directed against pre-identified targets may enhance treatment efficacy in PCCs/PGLs. In this regard, radiation treatment assays using MPC spheroids allow for evaluating the neo-adjuvant or radiosensitizing effects of potential candidate compounds. Since DMSO is frequently present as a solvent, its radioprotective effect on MPC spheroids has to be considered in drug testing studies.

## 4. Materials and Methods

### 4.1. Cell Lines and Routine Cultivation

Mouse pheochromocytoma cells (MPC) lacking endogenous HIF2α were recently genetically modified to express codon-optimized *Hif2α*, with gene expression being confirmed on the mRNA and protein level [27]. *Hif2α* expressing mouse pheochromocytoma (MPC) cells (MPC +HIF2α), as well as empty vector controls (MPC + EV) and wild-type (MPC^wt^) were routinely maintained in monolayer cell culture on collagen-coated flasks, as described elsewhere [27,41,54]. Cells were detached using trypsin and sub-cultivated as spheroids using a liquid overlay technique with nutritional medium containing 1 µg/mL penicillin and 1 µg/mL streptomycin (Life Technologies, Carlsbad, CA, USA) [27].

### 4.2. Microscopy

The size and morphology of the spheroids were routinely documented every 2 to 4 days using the fully automated differential interference contrast multi area time lapse tool of the Olympus FV12-548-295 confocal laser scanning microscope (Olympus, Tokio, Japan). For investigations involving incubation of spheroids with [^177^Lu]LuCl_3_, size and morphology were documented manually via bright field microscopy using the Microscope Axiovert 40 CFL (Carl Zeiss, Oberkochen, Germany).

Micrographs of the spheroids were routinely analyzed using Rover version 3.0.53h (ABX GmbH, Radeberg, Germany). The diameters of the spheroids were measured in regions of interest drawn via semi-automated contrast-based detection applying a threshold of between 0 and 180. In the case of contrast issues or large amounts of cellular debris, micrographs were analyzed manually using the polygon selection and fit spline tool of ImageJ version 1.51m (National Institute of Health, Bethesda, MD, USA), which was also applied for the measurement of circularity.

### 4.3. External Beam Radiation Treatment with X-ray

Spheroids were irradiated with a 200 kV photon beam using a Maxishot X-ray system equipped with a Y.TU/320-D03 tube (YXLON, Hamburg, Germany). Spheroids were treated with a single X-ray dose of between 0 to 40 Gy. The initial spheroid diameters (reached between 6 and 9 days after sub-cultivation), sample size, and number of experiments for each set up are summarized in Table 3, Tables S2 and S4.

### 4.4. Radionuclide Treatment with [^177^Lu]LuCl_3_

Lu-177 is a radionuclide emitting beta particles with a mean energy of 0.13 MeV, a maximum energy of 0.5 MeV, and an average range of 2 mm in water with a physical half-life of 6.7 days [55]. Spheroids were exposed to cell culture medium supplemented with [^177^Lu]LuCl_3_ (ITM Isotopen Technologien München, München, Germany; specific activity: no carrier added) at initial activity concentrations between 0 and 2 MBq/mL. Incubation with [^177^Lu]LuCl_3_ for 6 days approximately corresponded to an absorbed dose to water between 0 and 16 Gy. After 6 days, the incubation medium was replaced twice with fresh nutritional medium in order to remove most of the remaining [^177^Lu]LuCl_3_, giving the spheroids the chance to re-grow.

The energy dose D(t) [Gy = J/kg] approximately absorbed during the 6-day exposure of spheroids to [^177^Lu]LuCl_3_ was calculated from the initial activity concentration [MBq/mL] per well, as described elsewhere [32], using Equations (1) and (2), where A(t) is the total number of nuclear decays during the time of exposure T with the initial activity A_0_ and the physical half-life t_1/2_ of Lu-177 as a function of time describing the total number of beta particles, each emitted with the specific mean energy Ē of 0.13 MeV (1 MeV = 1.602∙10^−13^ J) into a well containing water-based liquid with a mass of m = 0.2 g.

(1) $$D\left(t\right)\;=\frac{∆E}{∆m}\;=\frac{A\left(t\right)\;\cdot \;\bar{E}}{m}$$

(2) $$A\left(t\right)\;=\int_{0}^{T}A_{0}\;\cdot \;0.5^{\frac{t}{t_{1/2}}}\;\mathrm{dt}$$

### 4.5. Statistical Analyses

Statistical analyses were performed using Prism 8 (GraphPad, La Jolla, CA, USA). All the data were visualized as mean ± SEM. If not stated otherwise, significance of differences was tested using analysis of variance applying Holm–Sidak’s post-hoc multiple comparison test.

### 4.6. Analyses of Treatment Effects on Spheroids

Data on spheroid diameters (d) monitored for up to 35 days after the treatment start were extracted from a series of micrographs and plotted individually. For evaluating the short-term effects of radiation treatments, changes in diameter within the initial 6 days after treatment start were determined, normalized to non-treated controls using Equation (3), and reported as relative spheroid growth (%SG). Relative spheroid growth values at increasing treatment doses were fitted with the equation for normalized one-phase decay (4). The corresponding growth arrest dose (GAD) was calculated at %SG = y = 0.

(3) $$\%\mathrm{SG}=\frac{d_{\mathrm{treatment},\;\mathrm{day}\;6}-d_{\mathrm{treatment},\;\mathrm{day}\;0}}{d_{\mathrm{control},\;\mathrm{day}\;6}\;-\;d_{\mathrm{control},\;\mathrm{day}\;0}}\;\cdot 100\%$$

(4) $$\%\mathrm{SG}=\left(100\%-P\right)\;\cdot e^{-\mathrm{kx}}+P$$

For evaluating the long-term effects of radiation treatments, the percentage of non-re-grown and therefore controlled spheroids per treatment group was monitored during a follow-up of up to 35 days after treatment start and reported as spheroid control probability (%SCP), as described elsewhere [52]. For each spheroid, an increase in diameter of less than 20 µm per day or a final diameter below 600 µm was defined as spheroid control. The 35-day %SCP values at increasing treatment doses were fitted with dose–response Equation (5) and the half-maximal spheroid control dose (SCD_50_) was calculated.

(5) $$\%\mathrm{SCP}=\frac{100\%}{{\left(\frac{{\mathrm{SCD}}_{50}}{x}\right)}^{h}+1}\;\;\;\;\;\;,\;h\;<\;20$$

## 5. Conclusions

The present study provides first experimental evidence that HIF2α-mediated pseudohypoxia contributes to radioresistance in PCCs/PGLs. The external irradiation and [^177^Lu]LuCl_3_ exposure of MPC spheroids provide surrogate models for radiation treatment and aid in investigating the metabolic and molecular determinants of radiation response in PCCs/PGLs. In particular, the effects of neo-adjuvant radiosensitizing treatments in combination with somatostatin type 2 receptor radiotherapy can be evaluated using these models.

## Figures and Tables

**Table 1:** Diameter changes within 3 days after treatment start (0–3 days) and between the third and sixth day of monitoring (3–6 days) displayed as growth rates.

Radiation Treatment		Diameter Changes after Treatment Start [µm/day]			
		MPC^wt^	MPC +HIF2α	MPC +EV	MPC +HIF2α
External X-ray irradiation					
**X-ray dose [Gy]**		**0–3 days**	**0–3 days**	**0–3 days**	**3–6 days**
0		47 ± 4	67 ± 2	49 ± 3	75 ± 2
4		−43 ± 2	34 ± 2	−24 ± 2	48 ± 2
8		−75 ± 1	19 ± 2	−39 ± 2	19 ± 1
12		−87 ± 2	16 ± 2	−54 ± 2	−3.4 ± 2
16		−89 ± 1	18 ± 2	−57 ± 1	−10 ± 2
20		−102 ± 2	11 ± 3	−52 ± 1	−22 ± 2
25		−108 ± 1	8.3 ± 3	−60 ± 2	−21 ± 2
40		−118 ± 1	1.7 ± 3	−66 ± 4	−26 ± 4
***r*_p_**		−0.84	−0.84	−0.72	−0.80
***p***		0.01	0.01	0.05	0.05
Incubation with [^177^Lu]LuCl_3_					
**Initial A*_V_*** **[MBq/mL]**	**(approx. β^−^ dose [Gy])**	**0–3 days**	**0–3 days**	**0–3 days**	**3–6 days**
0	(0)	*n. a.*	69 ± 2	52 ± 2	60 ± 3
0.03	(1)	*n. a.*	64 ± 2	24 ± 3	47 ± 4
0.05	(2)	*n. a.*	58 ± 2	−1.5 ± 3	20 ± 4
0.10	(4)	*n. a.*	49 ± 4	−6.7 ± 2	0.8 ± 6
0.15	(6)	*n. a.*	44 ± 5	−18 ± 2	−13 ± 13
0.25	(10)	*n. a.*	31 ± 2	−23 ± 1	−40 ± 5
***r*_p_**		*n. a.*	−0.97	−0.85	−0.99
***p***		*n. a.*	0.01	0.05	0.001

*r*_p_ Pearson’s linear correlation coefficient; *p* significance of linear relationship; *n.a.* not assessed.

**Table 2:** Growth arrest doses (GAD, 0–6 days after treatment start) and half-maximal spheroid control doses (SCD_50_, 0–35 days after treatment start) of MPC +HIFα and MPC +EV spheroids comparing external X-ray irradiation with [^177^Lu]LuCl_3_; diameters at treatment start: 492 ± 6 (MPC +HIF2α), 448 ± 3 (MPC + EV); significance of differences were calculated using *t*-test.

Treatment	Parameter	Measurand [Unit]	MPC + EV	MPC +HIF2α	*p*
X-ray	GAD	X-ray dose [Gy]	5.0 ± 0.3	16 ± 1.0	0.001
[^177^]LuCl_3_	GAD	Initial A*_V_* [MBq/mL]	0.3 ± 0.02	1.7 ± 0.7	0.05
[^177^]LuCl_3_	GAD	(approx. β^−^ dose [Gy])	(2.2 ± 0.2)	(14 ± 6.0)	
X-ray	SCD_50_	X-ray dose [Gy]	17 ± 0.2	21 ± 0.3	0.001
[^177^]LuCl_3_	SCD_50_	Initial A*_V_* [MBq/mL]	0.3 ± 0.02	0.6 ± 0.02	0.001
[^177^]LuCl_3_	SCD_50_	(approx. β^−^ dose [Gy])	(2.7 ± 0.1)	(4.6 ± 0.2)	

**Table 3:** Overview of sample sizes and average initial spheroid diameters in different investigations and data analyses.

Treatment	MPC^wt^		MPC + EV		MPC +HIF2α	
	*n*	*d* [µm]	*n*	*d* [µm]	*n*	*d* [µm]
X-ray	10	616 ± 2	10	479 ± 3	10	499 ± 3
[^177^Lu]LuCl_3_	*n. a.*	*n. a.*	20 ^†^	448 ± 3	20 ^†^	492 ± 6

† two independent experiments.

## Data Availability

The data presented in this study is contained within the article and supplementary material.

## Supplementary Materials

The following are available online at https://www.mdpi.com/2072-6694/13/3/385/s1: Figure S1: Circularity of irradiated MPC +HIF2α spheroids compared to MPC +EV spheroids after dose-dependent re-growth; Figure S2: Impact of G418 and DMSO on single-dose X-ray irradiation treatment of MPC +HIF2α and MPC +EV spheroids; Figure S3: Dependency of spheroid diameter changes on peripheral or central well positions in microplates; Figure S4: Impact of initial spheroid size on long-term response of MPC +HIF2α and MPC +EV spheroids to [^177^Lu]LuCl_3_ treatment; Table S1: Application of dose approximation on examples in literature; Table S2: Sample sizes and average initial diameters MPC spheroids analyzed for characterizing impact of G418 and DMSO supplements on X-ray treatment effects; Table S3: Impact of G418 or DMSO on the 6-day growth arrest dose (GAD) and half-maximal spheroid control dose (SCD_50_) after external X-ray irradiation; Table S4: Overview of sample sizes and average initial diameters of MPC spheroids analyzed for investigations on [^177^Lu]LuCl_3_ treatment effects at different initial spheroid size; Table S5: Half-maximal spheroid control doses (SCD_50_) of MPC spheroids in response to [^177^Lu]LuCl_3_ treatment at different initial diameters.

## Abbreviations

DMSO	dimethylsulfoxide
DOTA	1,4,7,10 tetraazacyclododecane-*N,N′,N″,N″′* tetraacetic acid
G418	geneticin
GAD	growth arrest dose
HIF	hypoxia-inducible factor
MPC	mouse pheochromocytoma cell line
PCCs/PGLs	pheochromocytomas and paragangliomas
SCD_50_	half-maximal spheroid control dose
%SCP	spheroid control probability
%SG	relative spheroid growth
SSTR2	somatostatin type 2 receptor
TATE	(Tyr^3^)octreotate
