# Supplementary material for: HIF2alpha-Associated Pseudohypoxia Promotes Radioresistance in Pheochromocytoma: Insights from 3D Models
Source: Cancers (Basel). 2021 Jan 21;13(3):385. doi: 10.3390/cancers13030385 (PMC7865577; doi:10.3390/cancers13030385)
Supplement: Supplementary file 1 [file cancers-13-00385-s001.pdf]

Supplementary Materials: HIF2alpha-Associated Pseudohypoxia Promotes Radioresistance in Pheochromocytoma: Insights from 3D Models

Verena Seifert, Susan Richter, Nicole Bechmann, Michael Bachmann, Christian G. Ziegler, Jens Pietzsch, and Martin Ullrich

1. References for approximation of absorbed β⁻ dose

Since there are no simulations for absorbed dose in spheroids seeded in concave-bottomed microtiter plates available yet, we approximated dose based on administered initial activity using equation 1 and 2. To confirm our calculations, we applied them to examples in literature of irradiated cells in monolayer culture.

Supplemental Table S1. Application of dose approximation on examples in literature.

Lit.	Nuclide	$t_{1/2}$ [h]	E_{avg} [MeV]	A_0 [MBq]	τ [h]	V [mL]	D_{Lit} [Gy]	D [Gy]	ΔD [%]
[32]	Y-90	64	0.935	100	1	2	21.7	26.8	24
	Re-188	17	0.765	100	1	2	18.8	21.6	15
	Re-188	17	0.765	4530	0,5	100	10	10	0
	I-131	192	0.182	100	1	2	5.6	5.2	7
	I-131	192	0.182	2	48	0.2	43	46	7
	I-131	192	0.182	4	1	4	0.114	0.105	8
[33]	Y-90	64	0.935	0.0048	192	0.022	1	1	0
	Y-90	64	0.935	0.048	192	0.022	10	10	0
	Y-90	64	0.935	0.0288	192	0.022	6.2	5.7	8
	Y-90	64	0.935	0.288	192	0.022	62	57	8

[32] Gholami, Y.H., et al., Comparison of radiobiological parameters for 90 Y radionuclide therapy (RNT) and external beam radiotherapy (EBRT) in vitro. EJNMMI physics, 2018. 5(1): p. 18.

[33] Freudenberg, R., et al., Dosimetry of cell-monolayers in multiwell plates. Nuklearmedizin, 2009. 48(03): p. 120-126.

$t_{1/2}$ Physical half-life of radionuclide

E_{avg} Mean energy of beta perticles

A_0 Initial activity

τ Exposure time

V Total volume for incubation

D_{Lit} Dose calculated in respective literature

D Dose approximated using equations 1 and 2

2. Spheroid circularity after re-growth

External beam X-ray irradiation affected morphology of regrown spheroids. Spheroid circularity showed a negative linear relationship with absorbed dose in MPC+HIF2α spheroids ($r = 0.853$, $p < 0.05$). Both MPC +HIF2α and MPC +EV spheroids showed significant differences ($p < 0.05$) in circularity between irradiated and non-treated control spheroids.

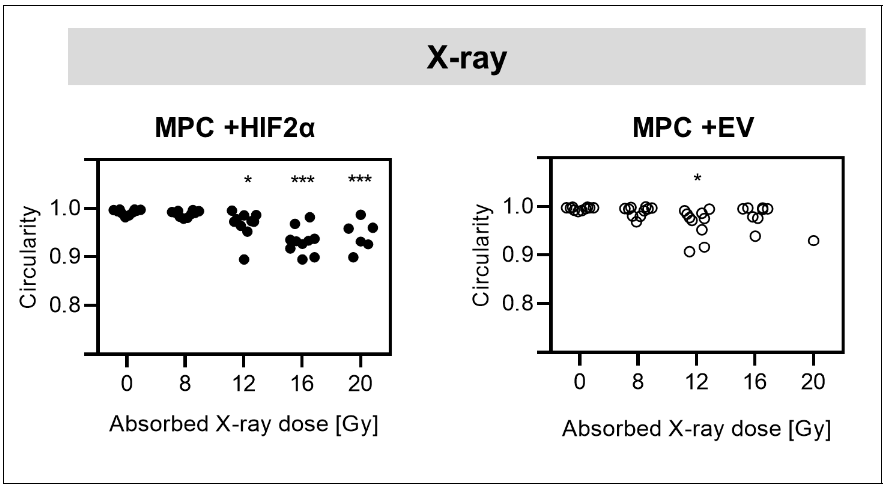

Supplemental Figure S1. Circularity of irradiated MPC +HIF2α spheroids compared to MPC +EV spheroids after dose-dependent re-growth; circularity was monitored at diameters of 670 ± 7 µm (MPC +EV spheroids treated with 20 Gy are not shown as they did not show re-growth to a diameter threshold of 600 µm); significant changes compared to non-treated spheroids (0 Gy), are displayed: (*) $p < 0.05$; (***) $p < 0.001$.

3. Impact of G418 and DMSO on external X-ray irradiation effects

Geneticin (G418) and dimethylsulfoxide (DMSO) are commonly used additives in cell culture studies. Here, we applied these agents during external X-ray irradiation of genetically modified MPC spheroids (Table S2). Supplementation with G418 or DMSO in concentrations of 250 µg/mL and 0.5%, respectively, influenced treatment outcome after external X-ray irradiation in MPC +HIF2α and MPC +EV spheroids. Especially dose-response to 20 and 25 Gy in *Hif2α* expressing MPC and to 16 and 20 Gy in empty vector controls was affected (Figure S2A). G418 significantly decreased spheroid control dose, whereas DMSO had radioprotective properties and the necessary doses for growth arrest and sustained spheroid control were increased (Table S3, Figure S2B).

Supplemental Table S2. Sample sizes and average initial diameters MPC spheroids analyzed for characterizing impact of G418 and DMSO supplements on X-ray treatment effects.

Supplement	**MPC +HIF2α**		**MPC +EV**	
	n	*d [µm]*	*n*	*d [µm]*
w/o	10	499 ± 3	10	479 ± 3
G418	17†	474 ± 2	17†	473 ± 2
DMSO	10	517 ± 6	10	479 ± 2
	ǯ	501 ± 3		

ǯ as 90% of MPC +HIF2α spheroids in the control group exceeded set diameter limits 400 – 555 µm with initial diameters > 600 µm marked diameter relates to average of samples excluding control group

† two independent experiments

Supplemental Table S3. Impact of G418 or DMSO on the 6-day growth arrest dose (GAD) and half-maximal spheroid control dose (SCD$_{50}$) after external X-ray irradiation; significance of differences was tested compared to treatment groups without additional supplement (*w/o*), respectively

	Supplement	**MPC +HIF2α**	*p*	**MPC +EV**	*p*
**GAD [Gy]**	*w/o*	16 ± 1		5.0 ± 0.3	
	+G418	19 ± 3	*n.s.*	5.2 ± 0.2	*n.s.*
	+DMSO	52 ± 38	*n.s.*	6.4 ± 0.3	0.01
**SCD$_{50}$ [Gy]**	*w/o*	21 ± 0.3		17 ± 0.2	
	+G418	18 ± 0.3	0.001	15 ± 0.3	0.01
	+DMSO	24 ± 0.1	0.001	22 ± 0.7	0.001

n.s. not significant

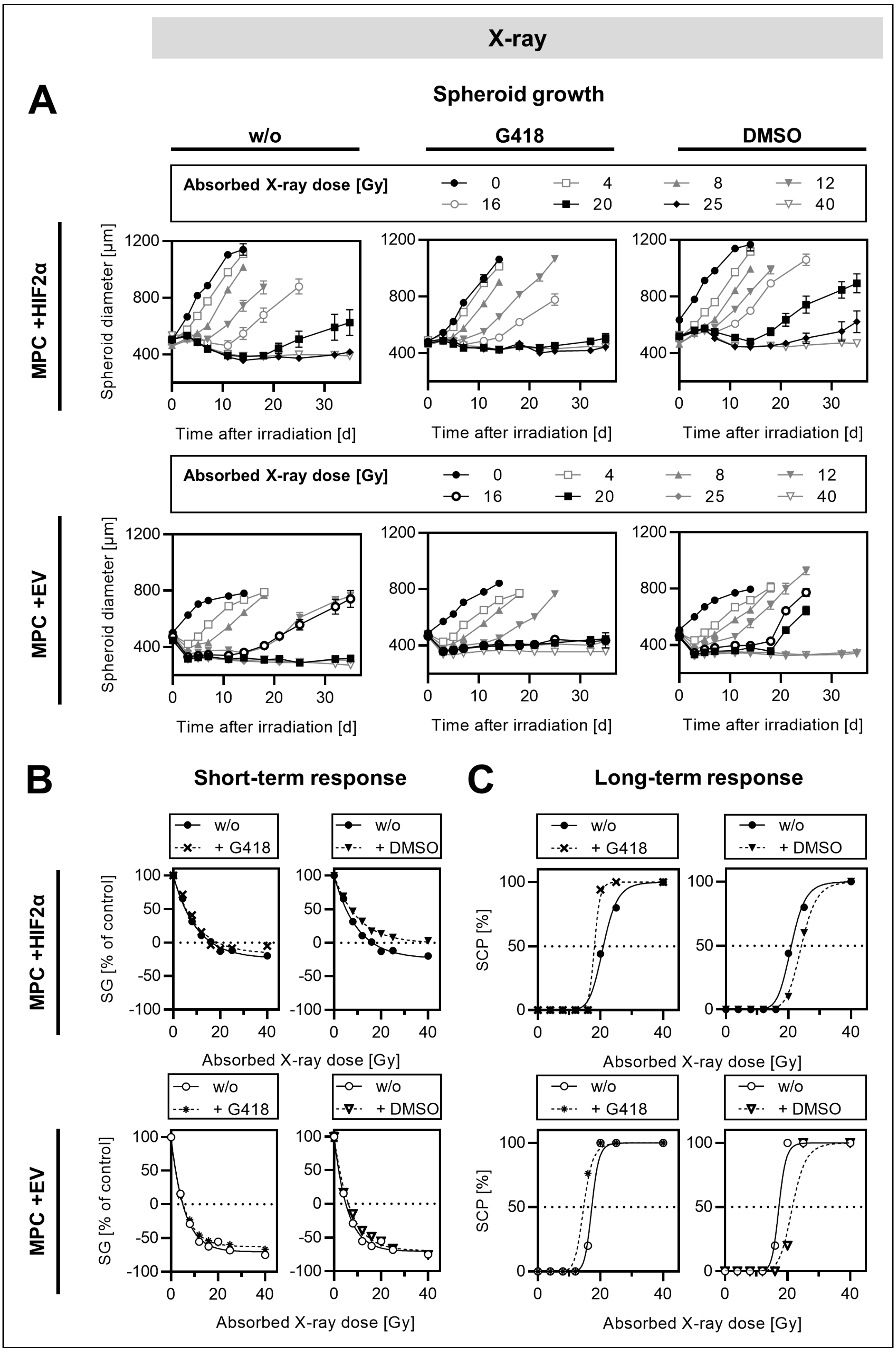

Supplemental Figure S2. Impact of G418 and DMSO on single-dose X-ray irradiation treatment of MPC +HIF2α and MPC +EV spheroids; (**A**) Monitoring of spheroid growth as diameter versus time plots, (**B**) 6-day dose response plotted as spheroid growth (%SG) versus absorbed X-ray irradiation dose; (**C**) 35-day dose response plotted as spheroid control probability (%SCP) versus absorbed X-ray irradiation dose.

4. Additional investigations on experimental set-up and plate layout for radionuclide treatment with [^{177}Lu]LuCl$_3$ *in vitro*

Lutetium-177 is a radionuclide emitting β^- particles and γ photons. In order to assure a reliable experimental set-up, it was mandatory to exclude interferences of radiation and absorbed dose between wells. Therefore, we scanned diameters of MPC^{wt} spheroids 6 days after treatment start in different plate layouts. These experiments showed no dependency of short-term response on spheroid positions in the microtiter plate (Figure S3).

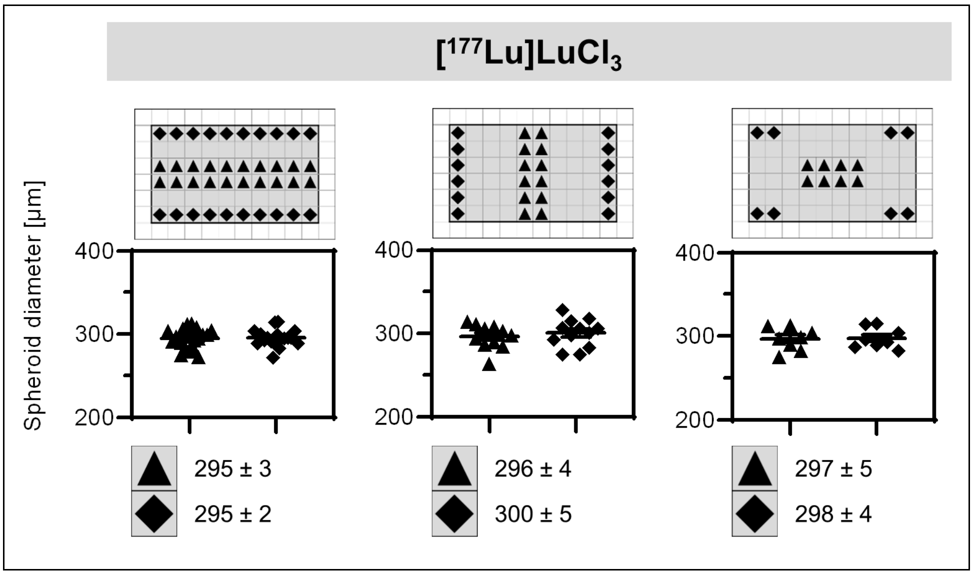

Supplemental Figure S3. Dependency of spheroid diameter changes on peripheral or central well positions in microplates; analyses of MPC^{wt} spheroid diameters after 6 days of [^{177}Lu]LuCl$_3$ treatment with initial activity concentration of 0.15 MBq/mL (≈ 1.2 Gy).

5. Impact of initial spheroid size on treatment effects of [^{177}Lu]$LuCl_3$

Since generating similarly sized spheroids in independent experiments can be challenging, we evaluated the impact of initial spheroid diameter on long-term response to incubation with [^{177}Lu]$LuCl_3$ in *Hif2α* expressing as well as empty vector control spheroids. Experimental parameters are summarized in Table S4. The dose necessary for sustained control of spheroids was significantly increased ($p < 0.001$) in larger spheroids and was overall significantly elevated ($p < 0.001$) in MPC +HIF2α compared to MPC +EV (Table S5, Figure S5A). SCD_{50} increased exponentially with initial spheroid diameter (Figure S5B).

Spheroid size was categorized in a cell-line-specific manner into four groups, based on measurements of initial diameters of more than 200 spheroids per cell line (Figure S5C): very small (< 370 μm), small (370 – 440/430 μm), large (440 – 590 / 430 – 570 μm), very large (> 590/570 μm).

In respect to these findings, we chose a medium diameter range of 400 to 555 μm for all herein reported irradiation experiments, as size-dependent differences of irradiation response are negligible for this size range, but necrotic cores and hence intrinsic hypoxia have already developed. Sustained control was not achieved for very large MPC +HIF2α spheroids, even with the highest activity concentration of 2 MBq/mL (≈16 Gy). These spheroids have large hypoxic areas enhancing with stabilization of HIF2α and thereby driving radioresistance in *Hif2α* expressing cells.

Supplementary Table S4. Overview of sample sizes and average initial diameters of MPC spheroids analyzed for investigations on [^{177}Lu]$LuCl_3$ treatment effects at different initial spheroid size.

	**MPC +HIF2α**		**MPC +EV**	
Diameter range [μm]	*n*	*d* [μm]	*n*	*d* [μm]
< 370	7	322 ± 4	5	314 ± 2
370 – 440/430	9	402 ± 2	9	404 ± 2
440 – 590 / 430 – 570	16	526 ± 3	16	474 ± 2
> 590/570	5	648 ± 4	5	626 ± 5

Supplementary Table S5. Half-maximal spheroid control doses (SCD_{50}) of MPC spheroids in response to [^{177}Lu]$LuCl_3$ treatment at different initial diameters.

**MPC +HIF2α**		**MPC +EV**	
Diameter range [μm]	SCD_{50} [MBq/mL] (approx. β^- dose [Gy])	Diameter range [μm]	SCD_{50} [MBq/mL] (approx. β^- dose [Gy])
< 370	0.35 ± 0.02 (2.85 ± 0.17)	< 370	*n.a.* † (*n.a.* †)
370 – 440	0.49 ± 0.003 (3.93 ± 0.02)	370 – 430	0.35 ± 0.02 (2.8 ± 0.1)
440 – 590	0.81 ± 0.02 (6.50 ± 0.10)	430 – 570	0.335 ± 0.01 (2.9 ± 0.10)
> 590	*n.a.* ‡ (*n.a.* ‡)	> 570	1.94 ± 0.02 (16 ± 0.4)

† could not be calculated on basis of available data but were below < 0.25 MBq/mL (1 Gy)

‡ could not be calculated on basis of available data but exceeded > 2 MBq/mL (16 Gy)

n.a. not assessed

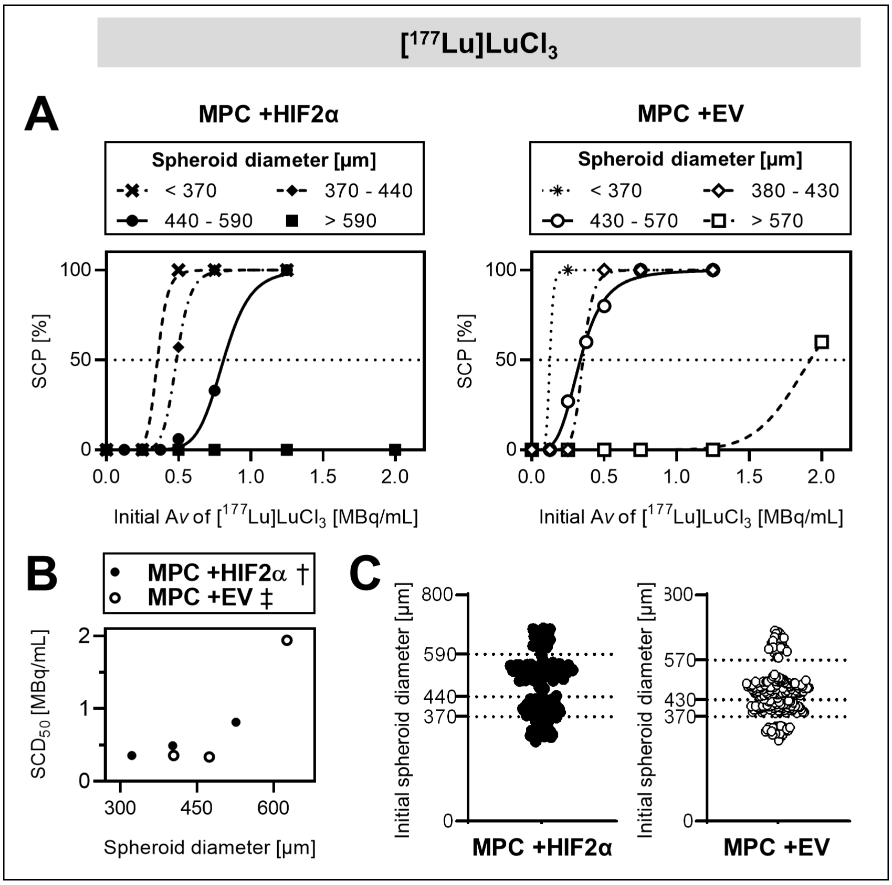

Supplemental Figure S4. Impact of initial spheroid size on long-term response of MPC +HIF2*α* and MPC +EV spheroids to [177Lu]LuCl3 treatment; (**A**) Spheroid control probability (%SCP) versus initial activity concentration of [177Lu]LuCl3; (**B**) Size-dependency of long-term response plotted as half-maximal spheroid control dose (SCD_{50}) versus initial spheroid diameters († SCD_{50} of MPC +HIF2*α* spheroids > 590 µm could not be calculated as SCP = 0 for all tested initial activity concentrations predicting an SCD_{50} > 2 MBq/mL; ‡ SCD_{50} for MPC +EV spheroids < 370 µm could not be calculated with available data); (**C**) Categorization of spheroids depending on initial diameter.

Publisher's Note: MDPI stays neutral with regard to jurisdictional claims in published maps and institutional affiliations.

© 2021 by the authors. Submitted for possible open access publication under the terms and conditions of the Creative Commons Attribution (CC BY) license (http://creativecommons.org/licenses/by/4.0/).
